# Optimal endobronchial tool sizes for targeting lung lesions based on 3D modeling

**DOI:** 10.1371/journal.pone.0189963

**Published:** 2017-12-19

**Authors:** Torre M. Bydlon, Gerrit C. Langhout, Ferry Lalezari, Koen J. Hartemink, Jasper Nijkamp, Susan G. Brouwer de Koning, Sjaak Burgers, Benno H. W. Hendriks, Theodoor J. M. Ruers

**Affiliations:** 1 Philips Research, 2 Canal Park, Third Floor, Cambridge, MA, United States of America; 2 Philips Research, High Tech Campus 34, AE Eindhoven, The Netherlands; 3 Department of Surgery, Netherlands Cancer Institute–Antoni van Leeuwenhoek Hospital, Plesmanlaan 121, CX Amsterdam, The Netherlands; 4 Department of Radiology, Netherlands Cancer Institute–Antoni van Leeuwenhoek Hospital, Plesmanlaan 121, CX Amsterdam, The Netherlands; 5 Department of Thoracic Oncology, Netherlands Cancer Institute–Antoni van Leeuwenhoek Hospital, Plesmanlaan 121, CX Amsterdam, The Netherlands; 6 Biomechanical Engineering Department, Technical University of Delft, Mekelweg 2, CD Delft, The Netherlands; 7 MIRA Institute, University of Twente, AE Enschede, The Netherlands; Texas A&M University, UNITED STATES

## Abstract

**Background:**

For patients with suspicious lung lesions found on chest x-ray or CT, endo/trans- bronchial biopsy of the lung is the preferred method for obtaining a diagnosis. With the addition of new screening programs, a higher number of patients will require diagnostic biopsy which will prove even more challenging due to the small size of lesions found with screening. There are many endobronchial tools available on the market today and a wide range of new tools under investigation to improve diagnostic yield. However, there is little information available about the optimal tool size required to reach the majority of lesions, especially peripheral ones. In this manuscript we investigate the percentage of lesions that can be reached for various diameter tools if the tools remain inside the airways (i.e. endobronchial biopsy) and the distance a tool must travel “off-road” (or outside of the airways) to reach all lesions.

**Methods and findings:**

To further understand the distribution of lung lesions with respect to airway sizes and distances from the airways, six 3D models of the lung were generated. The airways were modeled at two different respiratory phases (inspiration and expiration). Three sets of 1,000 lesions were randomly distributed throughout the lung for each respiratory phase. The simulations showed that the percentage of reachable lesions decreases with increasing tool diameter and decreasing lesion diameter. A 1mm diameter tool will reach <25% of 1cm lesions if it remains inside the airways. To reach all 1cm lesions this 1mm tool would have to navigate through the parenchyma up to 8.5mm. CT scans of 21 patient lesions confirm these results reasonably well.

**Conclusions:**

The smaller the tool diameter the more likely it will be able to reach a lung lesion, whether it be for diagnostic biopsy, ablation, or resection. However, even a 1mm tool is not small enough to reach the majority of small (1-2cm) lesions. Therefore, it is necessary for endobronchial tools to be able to navigate through the parenchyma to reach the majority of lesions.

## Introduction

For patients with suspicious lung lesions found on chest x-ray or CT, endobronchial and/or transbronchial biopsy of the lung is the preferred method for obtaining a diagnosis, with transthoracic CT-guided biopsy and surgical biopsy as alternative approaches. The NELSON study found that 62.7% of lesions were located in the periphery of the lung compared to the pleural wall (10%), middle (11.6%), or central (15.3%) airways [1]. In the central airways, approximately 4 endobronchial (tools remain in the airways) biopsies are needed to obtain an adequate diagnostic yield which has been reported to be 70–90% [2]. For peripheral lesions, fluoroscopy-guided transbronchial (tools traverse the airway wall) biopsy is the standard technique. Reported rates for diagnostic yield in peripheral lesions are 30–78% [2,3,4,5] and are especially low for small lesions <2cm in diameter (30–34%) [4,5]. Attempts have been made with endobronchial ultrasound (EBUS) probes, electromagnetic (EM) navigation, virtual navigation bronchoscopy, and bronchoscopic transparenchymal nodule access to improve diagnostic yield; however, none of these techniques have gained wide acceptance [6,7]. Overall ~60% of patients will return for a second procedure (transthoracic or surgical biopsy) if diagnostic yield is insufficient [3,6].

In the last decade several large population studies have been completed to assess the benefits of low-dose CT (LDCT) screening in the general population which has led to some countries implementing screening programs [8,9,10,11,12,13,14,15,16,17]. Given the increasing number of small, peripheral lesions detected by these screening programs, better biopsy tools will be required to achieve good diagnostic yield and follow-up.

There are many types of new lung tools that have been reported in the literature and some that are now entering the market. These new tools aim to improve diagnostic yield by providing better navigational guidance to the lesion (example, EM tracking [18,19,20]) or by evaluating the tissue prior to biopsy (examples, EBUS [21], radial endobronchial ultrasound [REBUS] [21], optical coherence tomography [22,23,24,25,26,27], fluorescence spectroscopy [28,29], diffuse reflectance spectroscopy [28,29,30], Raman spectroscopy [31,32,33], or differential path length spectroscopy [30,34,35,36]). These new tools will improve diagnostic yield but only if they can physically reach the lesion. After evaluating the current literature we were unable to find sufficient information on the ideal tool size required to be effective in targeting the majority of lung lesions endobronchially. The goal of this manuscript was to therefore investigate the percentage of lesions that can be reached for various diameter tools if the tools remain inside the airways (i.e. endobronchial biopsy) and the distance a tool must travel “off-road” (or outside of the airways) to reach all lesions.

## Methods

### 3D simulations of the human airways and randomly distributed lesions

A 3D model of the human airways, based on a deterministic algorithm that incorporates both duct branching and space division, has been developed by Hiroko Kitaoka et al. [37,38]. This model was used to generate an airway tree (Model Type 1) with 3311 branches. In the model the region of interest was set to 0 (whole lung), lung capacity at beginning of inspiration to 0.35, lung capacity at end of inspiration to 1.0, inspiratory time to 0.4, and a supine body posture assumed. For this exercise, only two time points in the respiratory cycle were used–when the lung is fully inflated (here forth referred to as ‘inspiration’) and when fully exhaled (‘expiration’). This model also generated a 3D volume of the entire lung.

**Fig 1:**
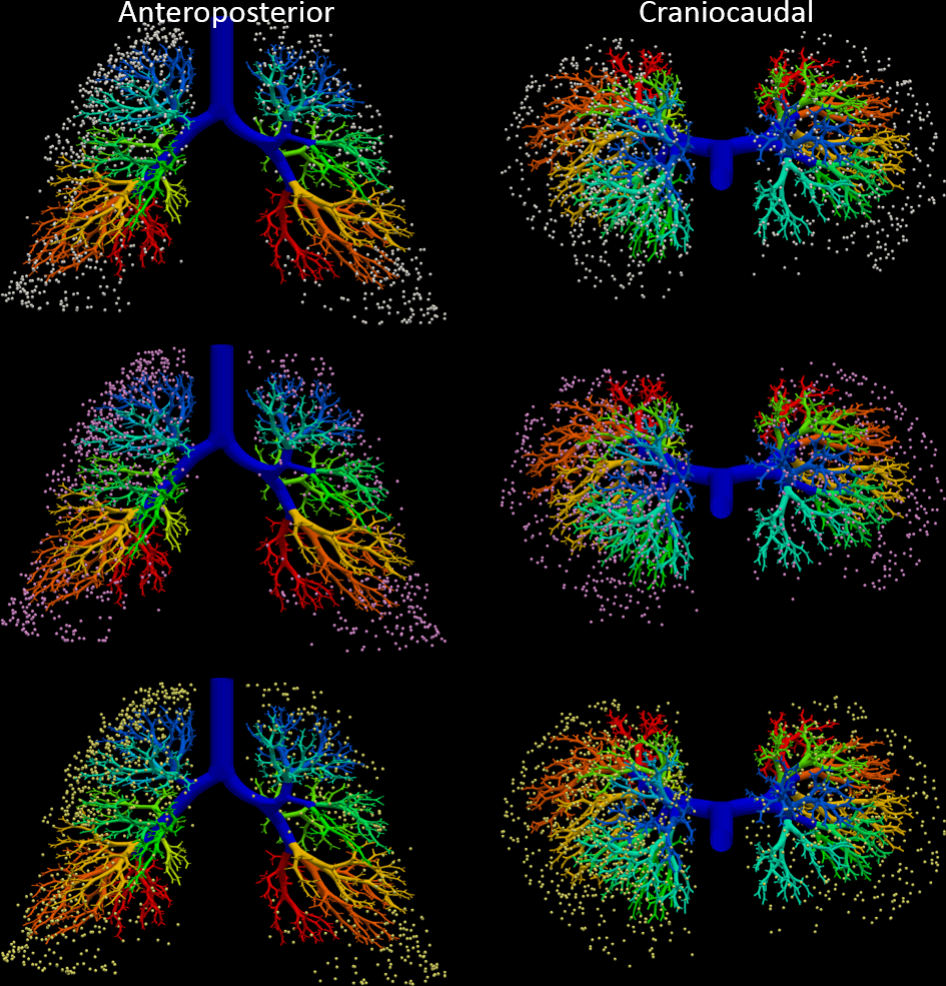
Simulated airway model at expiration showing 2mm lesions as spheres distributed randomly throughout the lung. The different colored lesions represent the 3 repeated models (white = model 1; pink = model 2; yellow = model 3). Images were created in ParaView 4.3.1.

Lung lesions were randomly generated using MATLAB (MathWorks, Natick, MA). Results from the NELSON trial [13] showed the distribution of lesions in the x-z (transverse) and x-y (coronal) planes. These distributions were used to define a probability distribution function (PDF) in the x- and y-axes; for the z-axis a uniform PDF was used for simplicity. A PDF was also defined for the left and right lung. The PDF’s were contained within the lung volume by manually selecting the minimum and maximum x, y, and z coordinates from the generated lung volume image; this was repeated 3 times to ensure the correct lung volume was used. The x, y, and z coordinates of 1000 points (i.e. lesions) were randomly drawn based on these PDF’s and 3 lung volumes. Three sets of lesions was drawn for the inspiration models and another 3 for the expiration models (in total 6 models of airways and lesions were created). A single set couldn’t be generated for both inspiration and expiration since the airways are in motion and the lung lesions are defined by fixed x, y, and z coordinates. The majority of lung lesions generated were found in the upper half of the lung or in the periphery, as expected based on the NELSON distributions. Fig 1 shows the expiration airway model with the three sets of 1000 randomly generated lung lesions.

The airway model is exported in VTK-format and consists of vertices and triangular faces. MATLAB was used to calculate the diameter of the airways at each face by iteratively stepping through each face, finding the opposite face, and calculating the distance between the two respective planes. When an opposite face could not be determined the diameter at that face was set to NaN (not a number).

Next, for each of the 1000 lesions in the 6 models (3 different distributions x 2 respiratory states), a spherical mesh was created to represent the lesion with diameters ranging from 1mm-5cm in 1mm increments. The *intriangulation* function in MATLAB was used to determine if there were any vertices or faces of the airway mesh that were inside the lesion mesh. For the faces and vertices that were found to be within the lesion mesh, the corresponding airway diameter was recorded. This was repeated for all lesion diameters, lesions, and models. To calculate the percentage of reachable lesions we counted the number of lesions that intersected with an airway whose diameter was larger than the tool size and divided by the total number of lesions (i.e. 1000); this percentage was calculated for tool sizes ranging from 1-9mm in 0.5mm increments.

**Fig 2:**
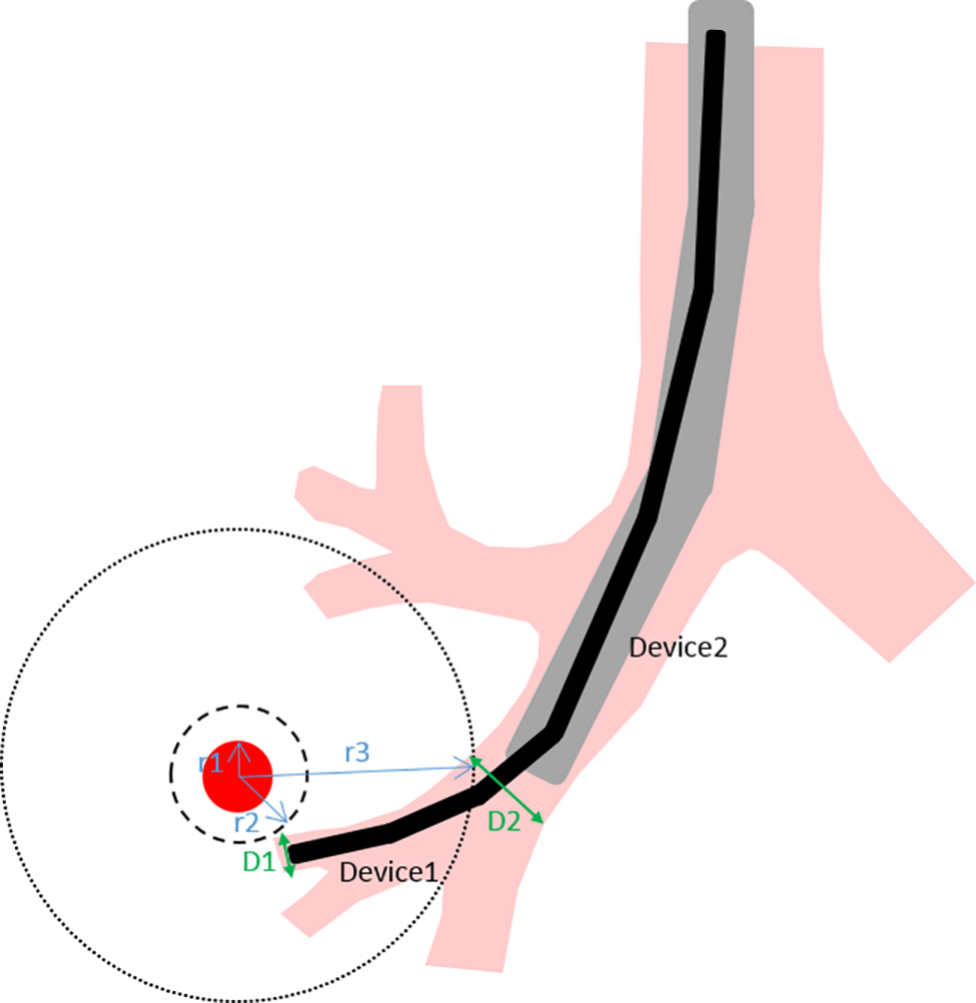
Depiction of how to calculate the off-road distance. The lesion is indicated by the red circle.

In these models, lesions are not always connected to an airway. Therefore, it is important to understand how far a tool must reach beyond the bronchial wall (i.e. going “off-road” in the parenchyma as in the case of transbronchial biopsy) to reach the boundary of a lesion. Fig 2 shows how this off-road distance was calculated. For example, given a lesion with radius r1, a small device (Device1) can be navigated inside the airways until it reaches an airway with diameter D1 which is approximately the same size as the tool itself. A larger device (Device2) can’t be navigated as far down the airways and gets stuck when the airway diameter is D2. At this point the tool would have to exit the airway and travel through the parenchyma to reach the lesion. In the case of Device1 this distance is simply the difference between r2 and r1, where r2 is taken as the radius of the first spherical lesion mesh which intersects with an airway greater than or equal to diameter D1. For Device2 the off-road distance would then be the difference between r3 and r1.

A few limitations should be noted regarding these simulations. First, the smallest airway diameter that is calculated from the Kitaoka airway tree is 0.1mm. If every branch termination was this small we could make conclusions about tool sizes down to 0.1mm. However, not every branch termination was smaller than 1mm, therefore, we will only draw conclusions based on tool diameters >1mm. Second, this analysis does not take into account any information about how the airways stretch or deform when a tool is navigated through them. Because the airways have some elasticity a tool may be able to be navigated into airways smaller than the tool diameter. Lastly, expiration and inspiration states were treated as two separate models with different lesion distributions and cannot be compared exactly.

### CT image analysis

This manuscript involves a retrospective analysis of CT images from lung cancer patients at the Netherlands Cancer Institute–Antoni van Leeuwenhoek Hospital. All patients underwent CT imaging as part of their standard clinical diagnostic care; no additional imaging examinations were performed for the data in this manuscript. The hospital ethics committee was consulted; under Dutch law the study did not require IRB approval (or patient informed consent). Only the authors associated with the hospital (GCL, FL) had access to the imaging data and performed the image analysis; measurement data was fully anonymized and de-identified. Images were restricted to patients with lung lesions >1cm and <4cm without prior radiotherapy. Helical CT Single Breath Hold Scans were reconstructed with 1.0mm slice thickness and 1.0mm slice increment. For lesions that were in contact with an airway, the airway diameter was measured. The distance between the lesion and closest airway of 1, 2, 4 and 6mm diameter was measured. Measurements were performed in the VUE PACS (Carestream Health, Rochester, NY, USA) viewing software using Cross-sectional MPR visualization.

## Results

**Fig 3:**
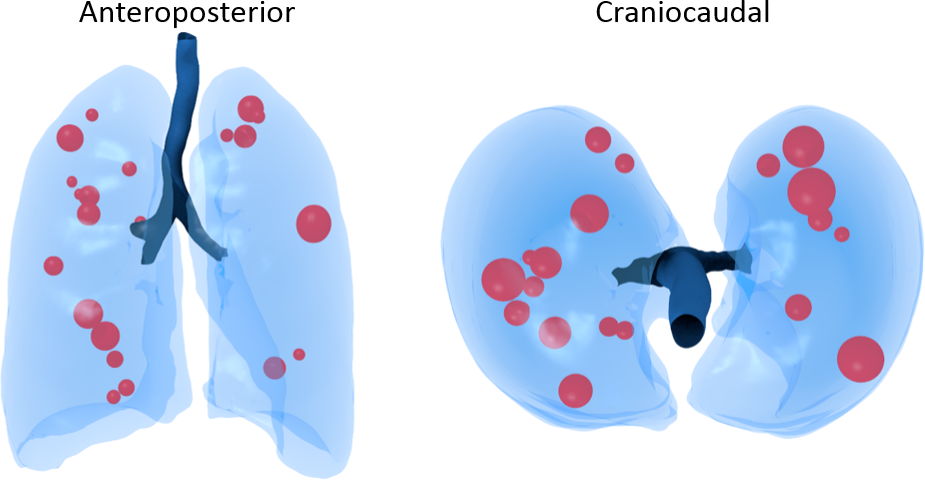
3D model of the lungs with the lesions (size and location) measured from the CT scans.

From the CT scans 21 lesions were included. The lesions were non-small cell lung carcinoma and sarcoma or melanoma metastases, ranging in diameter from 9.7mm to 3.6cm (1.6cm mean, 1.3cm median). Fig 3 shows the lesions projected in a 3D model derived from a CT scan without lesions: *isosurface* function in MATLAB and exported to Blender (Blender Foundation, Amsterdam, The Netherlands) for visualization. The size of the lesion represents the average diameters in the x, y and z direction. The location is the location of the measured lesion to the center of a 3D box enclosing the lungs. Most lesions appear in the right lung or upper lobes, similar to the NELSON trial [13] and simulated distributions.

**Fig 4:**
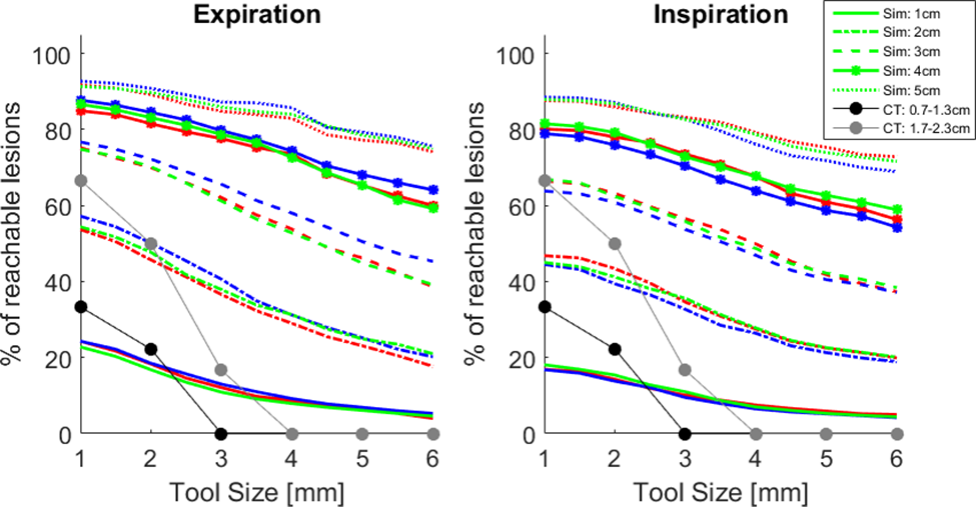
Percentage of lesions within reach of different sized tools if the tool remains inside the airways. Colors indicate the three simulated models (1- red, 2 –blue, 3 –green) and the type of line represents the targeted lesion diameter; black and gray are the CT data for lesions of 0.7–1.3cm and 1.7–2.3cm diameter respectively.

**Fig 5:**
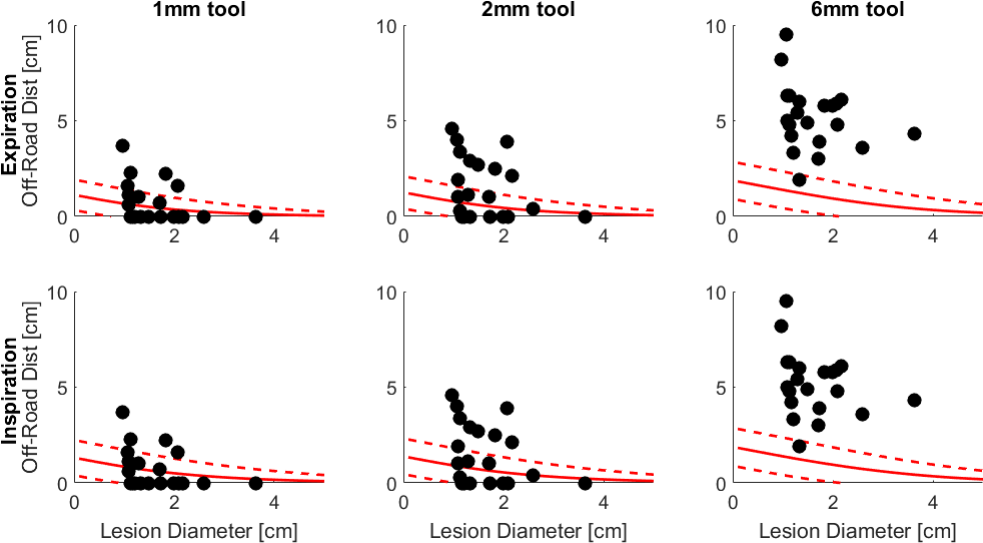
Distance a tool must travel off-road to reach a lesion in simulated model 1. The lines represent the average and standard deviations of the model. The black dots are the lesions measured from the CT scans.

In Figs 4 and 5 the simulated 3D data and the CT data are shown together for comparison. Fig 4 shows the percentage of reachable lesions versus various sized tools for different lesion sizes. Each of the 3 simulated models is shown as a separate color but for the most part these 3 models have very similar results. As would be expected, the smaller the tool size, the more lesions that can be reached. And the larger the lesion, the more likely it can be reached even with a larger tool. Although the number of lesions acquired from the CT scans is limited, there is reasonable agreement for the lesion sizes. In the CT data the majority of the lesions which touched an airway were next to 1 or 2mm airways; only one was next to a 3mm airway and one next to a 5mm airway.

Fig 5 shows the simulated average (and standard deviation) distance a tool would have to travel off-road to reach various sized lesions. Only one simulated model is shown since the other two models are nearly identical. For all tool sizes, the larger the lesion size the shorter the off-road distance. For example, a 6.3mm bronchoscope would have to go nearly 2cm to reach the smallest lesions, while a 1mm tool would only need to travel off-road ~1cm. The CT data overlaps reasonably well with the 1mm and 2mm diameter tools, however, diverges for the 6mm diameter tool.

## Discussion

The 3D simulations were used to better understand how small an endobronchial tool must be to reach the majority of lung lesions and how far a tool must travel beyond the bronchial wall through the parenchyma (off-road) if a lesion is not connected to the airways.

The simulations show that the percentage of small (<1cm) lesions that can be reached, even with a 1mm tool, is quite low at only 17.3% (inspiration) and 23.8% (expiration). To provide further insight into this, in one of the inspiration models 989/1000 1mm diameter lesions did not touch an airway; this only slightly decreases to 918/1000 for 5mm lesions. It is possible that these small lesions are touching an airway but they would be <1mm in diameter as this was the limit of our model. For the small lung lesions that will most likely be found with LDCT screening, this means that in endobronchial biopsy, the biopsy tool has to pass through the bronchiolar wall towards the lesion (transbronchial biopsy). For lesions 1cm in diameter, a 1mm tool would have to travel on average 8.5mm (inspiration) or 6.7mm (expiration) off-road, and farther for larger sized tools. To consider these results in another context, a standard 6.3mm diameter bronchoscope would reach <10% of 1cm lesions, <30% of 2cm lesions, and <50% of 3cm lesions. Most pulmonologists would agree that a bronchoscope this size can only reach central lesions which according to the NELSON study consisted of 15.3% of the overall lesions in the lung [1]; thus within the range of the percentages found here.

The CT data supports the results derived from the simulations; reasonable overlap is seen in both Figs 4 and 5. Compared to the 1000 simulated lesions in each model, the CT data is limited; with additional CT data it is assumed that the distributions in Fig 4 would match the simulated distributions more closely. Regardless of the data size, the CT lesions 0.7–1.3cm in diameter are close to the 1cm simulated curve, while the 1.7–2.3cm CT lesions are close to the 2cm simulated curve with the error between CT and simulation increasing with increasing tool size. On average, the off-road distance measured from the CT scans is close to the average calculated from the simulations for the 1mm and 2mm diameter tools, however, quite different for a 6mm tool. This is likely due to the fact that the airway diameters are slightly different between the simulated and CT data. From the CT scans the trachea measures ~1.8-2cm, the main bronchi ~1–1.4cm, and the lobar bronchi ~6-9mm. In the simulated data the trachea is ~1.4–1.5cm, the main bronchi ~1–1.2cm, and the lobar bronchi ~1–1.1cm; 6mm sizes do not occur until the segmental branches. The diameter difference in the lobar bronchi is likely the reason for the deviation between the CT and simulated data in both Figs 4 and 5. Since the lobar bronchi are larger in the simulated data, a large tool (i.e. 6mm bronchoscope) can be navigated further into the airways and hence the off-road distance to distal lesions is shorter compared to the CT data where a large tool will get stuck sooner. To confirm this assumption, we approximated the position of each CT nodule in the airway model and calculated the nearest airway diameter and off-road distance. The diameter of the closest airways were slightly larger when the CT nodules were placed in the airway model compared to the CT scans; and the off-road distance was smaller, especially for the 6mm diameter tool.

As was already mentioned in the Methods section there are some limitations with the models used in this analysis which may impact the conclusions that can be drawn from the results. First, our 3D lung model had 3311 branches. The smallest diameter airway in this model was 0.1mm, but this was not at the ends of every branch, therefore conclusions cannot be drawn for tools <1mm in diameter. Regardless, the ability to make a <1mm diameter tool with all the required functionality is almost impossible at this time and may therefore not be relevant.

The second, and likely more impactful, limitation of these models is that there is no stretching or deformation of the airways that is taken into account in determining the airway to lesion distances. Biological tissues have some elasticity and the airways will stretch when the endobronchial tools are advanced. Therefore, it is likely that in reality more lesions are reachable and the distance that the tools would need to travel is likely to be shorter.

It is also assumed that a tool traveling along an optimal path can be accurately navigated and localize any lesion. Obviously there are limitations to this today. However, with new techniques like electro-magnetic navigation bronchoscopy or image-guided bronchoscopy the ability to accurately navigate a tool along an optimal path may become more reasonable. Highly steerable, flexible tools would also be required for this.

Looking at the current literature and products on the market there are a variety of tools becoming available to pulmonologists to assess airways, biopsy tissue, and treat abnormalities. Given all of these new tools that could impact patient care it is important to understand how impactful they can be given their current sizes. Additionally for anyone designing new endobronchial tools it is important to understand the design criteria needed to target the majority of lung lesions.

Medtronic’s superDimension^™^ Navigation system and Veran Medical Technologies’ SPiN SYSTEM^™^ are probably the most widely known systems that are on the market to provide better navigational guidance, like a GPS, in the lung to localize lesions. superDimension^™^ consists of a tracking tool enabled with an electro-magnetic (EM) sensor and separate biopsy tools (1.7–1.9mm), like brushes, needles, forceps, etc., which are designed to fit down the working channel of a standard bronchoscope. From our simulations, the ~2mm EM sensor and biopsy tools would reach only 13.8–18.5% of 1cm lesions if the sensor remains in the airways. The benefit of a tracked technology is that the tools can still be located even when they must be used off-road. Overall, the clinical usefulness of EM navigational guidance is still under consideration. Some argue that it is a safe and effective tool for obtaining a diagnosis in high risk individuals that can’t undergo invasive procedures [18]. While other reports are less optimistic as diagnostic yield can be heavily impacted by a bronchus sign on CT and respiratory motion [19,20].

Ultrasound techniques, including linear endobronchial ultrasound (EBUS) and radial endobronchial ultrasound (REBUS) are beginning to play a larger role in the diagnosis and staging of lung malignancies by providing additional imaging of the airways [21]. Linear EBUS probes are typically quite large (>6mm diameter) and therefore restricted to the central airways. REBUS probes on the other hand, are much smaller (1.4 mm), potentially reaching more peripheral lesions. From the simulations a 1.4mm REBUS probe would directly reach 16.4–22.0% of 1cm lesions. It should be noted however that REBUS has a penetration depth of a few centimeters and would therefore be able to image lesions beyond the airway wall; thus the percentage of “reachable” lesions would be at least 4x higher than a tool that needs to be in direct contact with the tissue–which is the case for a biopsy. Although REBUS can potentially image more lesions than EBUS, the probe must be exchanged with forceps or a needle to take the actual tissue sample as it’s too large for the working channel to accommodate both tools.

A variety of optical based techniques can be found in the literature where they are predominately used to image the structure of the airways, like ultrasound, or to provide real-time biochemical and morphological information related to tissue composition. Light-based techniques are an attractive option because they are non-destructive to the tissue and are compact enough to fit through the working channel of a bronchoscope [22]. Tissue characterization tools like Raman spectroscopy [31,32,33], diffuse reflectance spectroscopy (DRS) [28,29,30], fluorescence spectroscopy [28,29], and differential path-length spectroscopy (DPS) [30,34,35,36] have been investigated for lung cancer detection, achieving >92% sensitivity and specificity with Raman [31,32] and >80% sensitivity and specificity with DRS and DPS [28,29,30,39]. These technologies have not yet been investigated for endobronchial access and it is unknown how small these types of tools could be made. Like ultrasound, they can also measure tissue beyond the airway wall with a penetration depth on the order of millimeters, thus increasing the percentage of reachable lesions 2-fold. Another optical technique, autofluorescence bronchoscopy, is already on the market and shows improved performance in sensitivity but is still limited to the central airways due to its size and specificity is low [31]. To our knowledge the only group that has investigated the feasibility of peripheral airway optical sensing is that of Suter et al. [23,24,26,27]. They developed an optical coherence tomography (OCT) imaging catheter integrated inside a 21-gauge TBNA needle to target peripheral airways which was tested in *ex-vivo* swine lungs [23]. OCT uses light scattering to produce an image of the tissue structure, similar to ultrasound but with much finer resolution and at the expense of imaging depth. The integration of both the imaging and biopsy needle into a single catheter shows real hope that a fully integrated tool could eventually be made. From our simulations, their 21-gauge needle (assuming a 2mm penetration depth) would reach ~28–36% of 1cm lesions.

From the 3D simulations and CT analyses, we have shown that for most lung lesions that will be detected with LDCT screening the small size of these lesions will make it challenging to reach, biopsy, and treat them endobronchially. In the majority of patients biopsy tools and/or treatment devices such as ablation needles will need to traverse the bronchial walls and proceed off-road to successfully reach the lesions; or we need tools that are <1mm in diameter–a challenging task. Typically the tool tip will need to travel 1-2cm off-road to reach and biopsy/treat a lesion. Given this information, any new technology being developed for navigational guidance, tissue confirmation, or treatment needs to be made small enough and should be able to traverse the bronchial wall to have the greatest impact in the pulmonology workflow.

## Supporting information

S1 File Matlab (.mat) file containing the x, y, and z coordinates of the simulated lesion locations with respect to the airway models. Inspiration and expiration are two different variables. Each variable contains 3 cells corresponding to the 3 models.
(MAT)

S2 File Excel file containing the airway diameters, lesion diameters, and distances to airways from the CT scans. (XLSX)

## Abbreviations

DPS: Differential path-length spectroscopy
DRS: Diffuse reflectance spectroscopy
EBUS: Endobronchial ultrasound
EM: Electromagnetic
LDCT: Low dose CT
NaN: Not a number
OCT: Optical coherence tomography
PDF: Probability distribution function
REBUS: Radial endobronchial ultrasound
TBNA: Transbronchial needle aspiration
